# Effects of Different Expansion Temperatures on the Non-Volatile Qualities of Tea Stems

**DOI:** 10.3390/foods13030398

**Published:** 2024-01-26

**Authors:** Xin Wang, Changxu He, Leyin Cui, Zhengquan Liu, Jin Liang

**Affiliations:** 1 State Key Laboratory of Tea Plant Biology and Utilization, School of Tea & Food Science and Technology, Anhui Agricultural University, Hefei 230036, China; 2 Key Laboratory of Jianghuai Agricultural Product Fine Processing and Resource Utilization, Ministry of Agriculture and Rural Affairs, Anhui Engineering Research Center for High Value Utilization of Characteristic Agricultural Products, College of Tea & Food Science and Technology, Anhui Agricultural University, Hefei 230036, China

**Keywords:** tea stems, expansion, temperature, metabolomics

## Abstract

Tea stems are a type of tea by-product, and a considerable amount of them is discarded during picking, with their value often being overlooked. To enhance the utilization of tea stems, we investigated the effects of different expansion temperatures on the non-volatile compounds of tea stems. The results showed that the contents of EC, EGC, EGCG, tea polyphenols, and amino acids all decreased with the expansion temperature, while the contents of GA and C increased. The best effect was observed at 220 °C for 20 s. Additionally, as the temperature increased, the umami and aftertaste of astringency values of tea stems decreased, and the value of bitterness increased. Meanwhile, the value of sweetness decreased first and then increased. EGC was identified as the key differential compound of tea stems at different temperatures. In this investigation, determining the optimum expansion temperature was deemed advantageous for enhancing the flavor quality of tea stems, consequently elevating the utilization efficacy of tea stems and tea by-products.

## 1. Introduction

Tea (*Camellia sinensis*), originating in China, is one of the most popular beverages globally [1]. It is widely known for its distinctive flavor and recognized efficacy [2,3]. Moreover, its biological activities are thought to be mainly related to *L*-theanine, caffeine, flavan-3-ol, and polysaccharides [4,5,6]. However, processing a large amount of fresh tea leaves into high-quality tea and liquid tea beverages has led to the simultaneous disposal of many tea by-products, such as low-grade tea, tea powder, tea residue, and tea stems, resulting in a certain degree of waste [7].

Tea stems are a leaf stalk plucked from finished tea, constituting approximately 35% of the dry weight of tea leaves [2]. They primarily contain tea polyphenols, tea polysaccharides, amino acids, alkaloids, aromatic compounds, and other active ingredients. Especially, the contents of catechins, tea polyphenols, water extracts, and caffeine of oolong tea stems were lower than that in oolong tea leaf, while the amino acids and total sugar content were higher [8]. Its physical structure is also different from that of tea, as the inner part of tea stem is loose and porous, thus having a better leaching effect and faster leaching speed after expansion.

In general, whether in the process of tea picking or processing, tea stems are usually discarded. However, tea stems develop a unique roasted and sweet aroma after high-temperature roasting [9], characterized by compounds such as pyrazines, pyrroles, and furans [10], accompanied by a change in color. In addition, related reports showed that high temperatures could reduce the content of tea polyphenols, catechins, and amino acids. Amino acids and sugars in tea stems could generate baking flavor compounds through the Maillard reaction, including pyrazines and pyrroles [11]. Therefore, improvement in the flavor of tea stems by high temperature could be applied in the production of tea beverages, drawing attention to the potential resources of tea stems and contributing to their value while minimizing waste. The principal aim of this experiment was to explore the effects of different expansion temperatures on the quality of tea stems, including tea polyphenols, caffeine, and other compounds. This was achieved through a combination of targeted and non-targeted metabolomics and electronic tongue analyses. The findings aim to serve as a reference for the future high-value utilization of tea stems, transforming them from by-products into valuable resources, promoting the rational utilization of resources and reducing resource waste.

## 2. Materials and Methods

### 2.1. Chemicals

Formic acid, methanol, and acetonitrile (liquid chromatography–mass spectrometry (LC-MS) grade) were purchased from Thermo Fisher Scientific (Fair Lawn, NJ, USA). Standards including gallic acid (GA, >98%), (+)-catechin (C, >98%), caffeine (CAF, >98%), (−)-epigallocatechin (EGC, >98%), (−)-epicatechin (EC, >98%), (−)-gallocatechin gallate (GCG, >98%), (−)-gallocatechin (GC, >98%), (−)-epicatechin gallate (ECG, >98%), and (−)-epigallocatechin gallate (EGCG, >98%) were purchased from Chengdu Must Bio-Technology (Chengdu, China). Folin–Ciocalteu’s phenol reagent was purchased from Solarbio Science & Technology Co., Ltd. (Beijing, China). DL-4-chlorophenylalanine was obtained from MedChemExpress (Shanghai, China). Ethylenediaminetetraacetic acid disodium (EDTA-2Na) was purchased from Sinop harm Chemical Reagent (Beijing, China). All other solvents and chemicals used in the tests were of analytical grade. The distilled water used in this study was obtained from Watsons Water Co. (Guangzhou, China).

### 2.2. Processing of Tea Stem Sample

Samples of tea stems were obtained from Hangzhou Saina Tea Co., Ltd. (Hangzhou, China). Steamed green tea stems (containing a small amount of leaves, hereafter referred to as tea stems) were assessed, and the tenderness of the tea stems was high (moisture content 92.44%). In the experimental process, a certain amount of quartz sand was added to the frying machine (operating at a speed of 15 r/min, Model 5 Computerized Fryer, Changzhou, China) for preheating. After reaching the setting temperature, tea stems were added into the preheated environment. Then, 20 s later, all the tea stems in the frying machine would be poured out to pass through the sieve and cooled down immediately. The tea stems were puffed at different expansion temperatures for 20 s: insufficient expansion group (180 °C/20 s, 200 °C/20 s), moderate expansion group (220 °C/20 s), and over expansion group (230 °C/20 s, 250 °C/20 s). All tea stem samples were stored at −20 °C before analysis. The following samples are referred to as the tea stems (CK), 180 °C, 200 °C, 220 °C, 230 °C, 250 °C.

### 2.3. Non-Volatile Quality Analysis of Tea Stems

#### 2.3.1. Quantitative Assessment of Tea Infusion Flavor by Electronic Tongue

The electronic tongue (TS-5000Z, INSENT, Tokyo, Japan) was used for testing. Tea infusion was extracted according to GB/T 23-776-2018 [12]. We retrieved a 35 mL sample of tea infusion into a testing cup to test the bitterness, umami, sweetness, richness, and astringency of the tea infusion when it cooled to room temperature, whilst making sure that the sensor and probe were not submerged in the liquid level and standard solution. Referring to the method of Wei et al. [13], each sample was collected four times, the first set of detection data were removed, and the next three sets were retained as detection results. The response value of the electronic tongue was obtained from the membrane potential difference between the reference electrode and the sensor, and then the potential value was converted to the taste value according to Weber’s Fechner’s law (the contrast between the perceived intensity and the stimulus intensity is proportional).

#### 2.3.2. Determination of Tea Polyphenols, Caffeine, and Catechins

The quantification of caffeine and catechins was determined by using an Agilent high performance liquid chromatograph (Agilent Technologies, Palo Alto, CA, USA) equipped with an Agilent SB-Aq C_18_ reversed-phase column (250 × 4.6 mm, 5 μm), protected by a Phenomeex Synergi Hydro-RP C_18_ HPLC guard column (10 × 4.6 mm^2^, 5 µm). Mobile phase A was 0.2% EDTA-2Na + 9% acetonitrile + 2% glacial acetic acid; mobile phase B was 80% acetonitrile + 2% glacial acetic acid + 0.2% EDTA-2Na. The linear gradient elution program was as follows: 0~10 min, 100% A; 10~25 min, 100~68% A; 0~32% B; 25~35 min, 68% A; 32% B; 35~40 min, 68~100% A; 32~0% B; 40~60 min, 100% A. Detection of caffeine and catechins was carried out based on the method of Ning [14], and the samples were slightly modified. Specifically, 0.2 g of tea stem powder (prepared with a grinder and sieved through a 20-mesh sieve) underwent two extractions with 5 mL of 70% methanol, obtaining 10 mL of the solution for analysis. The acquired solution was filtered through a 0.22 µm nylon membrane and analyzed by high performance liquid chromatography. The concentrations (mg/g) were then determined using standard curves for caffeine, EC, EGC, EGCG, C, ECG, GCG, GC, and GA.

The determination of tea polyphenols (TPs) was carried out in accordance with the standard GB/T 8313-2018 [15]. Firstly, 1 mL of the catechin test solution was diluted 100 times. Then, 5 mL of 1 mL of the mother liquor Folin–Ciocalteu’s phenol reagent was mixed into 1mL of the diluted solution. After reacting the solution with the reagent for 5 min, 4 mL of 7.5% Na_2_CO_3_ solution was added; then, water was added at 10 mL, shaken well, and then let to stand at room temperature for 60 min. Finally, absorbance was quantified at 765 nm by using a Hitachi U-5100 UV spectrophotometer (Tokyo, Japan).

#### 2.3.3. Sample Preparation for LC–Orbitrap–MS Analysis

Sample extraction and metabolomics analysis were referred to from the previous study by Wei et al. [16]. We accurately weighed 0.2 g of tea stem powder in a 10 mL centrifuge tube and added 4 mL of 70% methanol solution containing DL-4-chlorophenylalanine (200 mg/L) as an internal standard for sonication (40 kHz, 30 min, Model Shumei KQ-500DE, Kunshan, China); then, it was left at room temperature for 4 h. We then took out the centrifuge tube and shook it up and down evenly. After that, ultrasonic extraction was performed again, and then the solution was left to stand for a total of 8 h. Following centrifugation, 100 μL of supernatant was extracted and combined with 3 mL of a 70% methanol solution. Lastly, the 0.1 mL centrifuged supernatant was diluted 40 times and passed through a 0.22 μm nylon membrane for mass spectrometry. The extraction was repeated three times for each sample. Quality control (QC) samples were also prepared by mixing equal volumes of each test sample. The samples were stored at −20 °C before the test.

### 2.4. Data Analysis

The results from routine tests are presented as mean ± standard deviation. Each sample test was repeated three times and analyzed for variance using SPSS 25.0 (IBM, Armonk, NY, USA). *p* < 0.05 was considered significantly different. A one-way analysis of variance (ANOVA) was performed using Duncan’s test on SPSS. Heatmaps were created using TBtools (JRE version 1.6, Guangzhou, China). Bar graphs were made using Origin (version 2021).

The raw LC-MS datasets were converted to mz/ML and .abf format in software using MS-Convert and Analysis Base File Converter, respectively. The mass spectrometry data were then preprocessed with MS-DIAL and imported into SIMCA for diversified analysis. In SIMCA (version 14.1, Umetrics, Umea, Sweden), Centered, UV and Pareto could be selected for normalized data analysis, and the Pareto method was chosen to normalize the variables in this experiment, so as to reduce the influence of human factors and machine noise. MS-FINDER (version 3.04) was employed to analyze and compare the obtained *m*/*z* values, retention time, and ion fragmentation data with established standard databases, public metabolomic databases (ChEBI, UNDP, GNPS, and PubChem databases), and metabolite identification references.

## 3. Results and Discussion

### 3.1. Results of Electronic Tongue Detection with Different Temperature of Expansion

**Figure 1:**
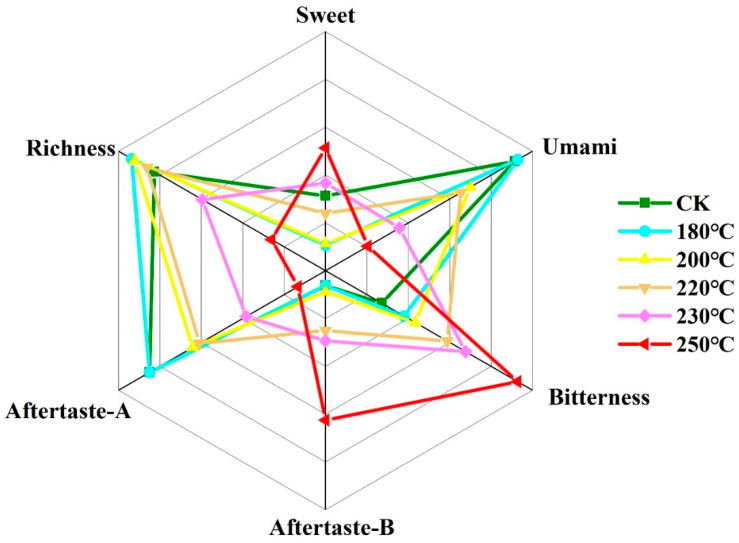
Electronic tongue radar map of tea stems with different expansion temperatures.

Traditional sensory evaluation, involving the assessment of tea attributes such as shape, color, aroma, and the taste of tea infusion, was typically conducted by professional tea evaluators [17]. This process possesses a certain degree of subjectivity. The limitations of sensory evaluation could be addressed through the utilization of the electronic tongue to compensate for the shortcomings of subjectivity, unpredictability, and inconsistency in the evaluation of tea [18]. The response value of the electronic tongue was obtained from the membrane potential difference between the reference electrode and the sensor. The electronic tongue test could datarize the traditional sensory review and made the results of sensory evaluation more scientific. The sourness and astringency of all the samples in this experiment were lower than the tasteless point (Table 1). In addition, it could be seen from Figure 1 that the sweetness decreased first and then increased with the increase in expansion temperature. The first half of the first decrease might be due to the fact that the rising temperature made the Maillard reaction produced by amino acids and sugars proceed rapidly, resulting in a decrease in the sweetness of the tea infusion. When the temperature was too high, the macromolecular compounds in the tea stems that were insoluble in water began to be degraded. For example, large molecules such as starch, cellulose, and proteins hydrolyze to produce amino acids, glucose, fructose, and other compounds [19], so that the sweetness of the tea infusion was increased. On the other hand, tea infusion umami decreased with the increase in expansion temperature, and it could be seen that from 200 °C onwards, umami decreased more quickly. Previous research has indicated that the reduction in amino acids has lead to a decrease in umami [3,19]. The richness of tea infusion first increased and then decreased, and it decreased significantly after 230 °C. This phenomenon might be attributed to the transformation of taste compounds in tea stems into aromatic compounds at elevated temperatures. With the increase in temperature, the bitterness of the samples gradually increased, and there was a significant difference between each group. The aftertaste of bitterness values showed a negative value in the tea stems (CK) and 180 °C, but the overall trend was positively correlated with the bitterness. As is commonly acknowledged, during the tea production process, high temperatures cause tea with a higher tenderness to burn and have a bitter taste in the tea infusion, accompanied by the appearance of burnt and red edges. Consequently, heightened temperatures correlated positively with the heightened prominence of burnt bitterness in tea stems. The gradual reduction in aftertaste of astringency values was attributed to the decline in astringent compounds, including flavanols (catechins), phenolic acids (theogallin), and flavanones [19]. Therefore, in the process of increasing the temperature of expansion, sweetness first decreased and then increased; richness demonstrated an initial increase and subsequently decreased; bitterness and the aftertaste of bitterness increased; and umami and the aftertaste of astringency decreased.

### 3.2. Content Analysis of Catechins, TPs, and Caffeine

The catechins in tea accounted for about 60–80% of the polyphenols in tea [20]. The catechins and EGCG were the source of bitterness and astringency [21], which played an important role in the flavor composition of tea. In contrast, the content of tea polyphenols, caffeine, and catechins in the tea stems was lower than that in the tea leaves [22]. Catechins could be categorized into two groups based on their conformation: *epi*-type catechins (EC, ECG, EGCG, EGC) and non-*epi* type catechins (GCG, C, CG, GC).

**Figure 2:**
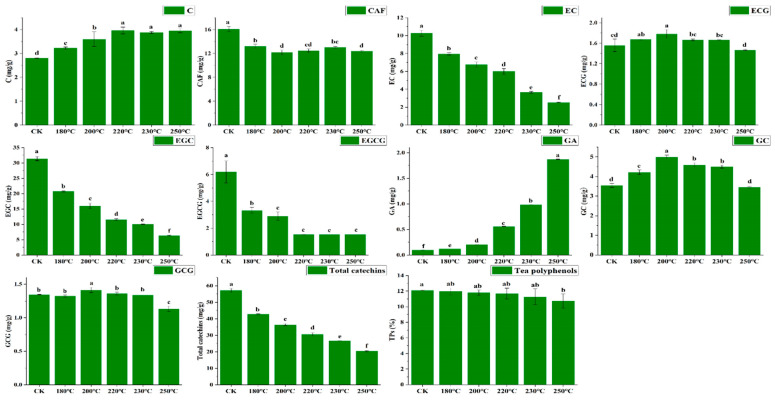
Catechins and tea polyphenols of tea stems at different expansion temperatures (*p* < 0.05). GA, gallic acid; GC, (−)-gallocatechin; EGC, (−)-epigallocatechin; C, (+)-catechin; CAF, caffeine; EGCG, (−)-epigallocatechin gallate; EC, (−)-epicatechin; GCG, (−)-gallocatechin gallate; TPs, total polyphenols. Different lowercase letters marked on the same row represent statistically significant differences in the data (*p* < 0.05).

The contents of EC, EGC, EGCG, and caffeine in the tea stems (CK) were significantly higher compared to the puffed samples (Table 2). Those also decreased between the puffed samples with the increase in expansion temperature. Among them, the trends in EC and EGC were significantly different between each expansion gradient. The EC, EGC, and EGCG in tea stems all decreased with increasing temperature (Figure 2), which was also similar to the findings in previous studies. It was shown that the *epi* type of catechins were isomerized under high-temperature conditions [10,23], and the content of ECG exhibited a trend of initial increase and then decrease with increasing temperature. During the high-temperature process, flavan-3-ols were replaced by N-ethyl-2-pyrrole gradually, for example EGCG [24], which in turn produced baking compounds and an increase in aroma concentration in the tea stems. As the expansion temperature increased, the contents of GA and C increased gradually, and the contents of tea stems (CK) were significantly lower than those in the puffed samples. The contents of GC and GCG were first increased and then decreased. Furthermore, the concentrations of tea polyphenols, caffeine, and total catechins exhibited a decrease (Figure 2), consistent with findings from earlier studies [21,25]. The concentration of tea polyphenols in tea stems (CK) was 12.1 ± 0.03%. However, the content of tea polyphenols gradually decreased with the increase in expansion temperature, which may have been due to the oxidative decomposition and polymerization of tea polyphenols by high temperature [26].

EGC, caffeine, and EC accounted for a larger proportion of the tea stems in this study. All three compounds were significantly reduced after expansion, which indicated that the main secondary metabolites in tea stems were changed after expansion. Especially, the content of EGC and EC decreased about five times after expansion.

### 3.3. Metabolomics Analysis of Changes in the Expansion Process of Tea Stems

In order to further understand the effect of different temperatures on the contents of tea stems, LC-Orbitrap-MS was used to perform non-targeted metabolomics analysis on different experimental samples. After peak extraction, alignment, and filtration, 4228 ion features were detected in the tea stem samples.

**Figure 3:**
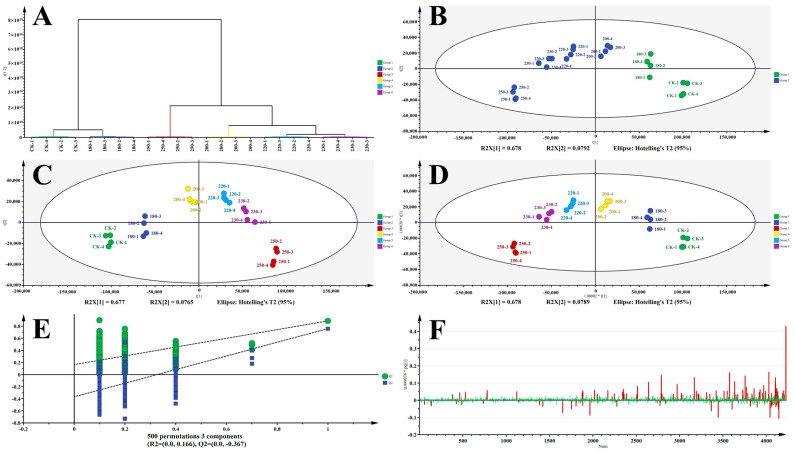
Multivariate analysis of LC-MS based metabolomics data of tea stems with different expansion temperature; (**A**) clustering analysis (HCA); (**B**) score plots of principle component analysis (PCA); (**C**) partial least squares data analysis (PLS-DA); (**D**) orthogonal partial least squares data analysis (OPLS-DA); (**E**) permutation test result of OPLS-DA; (**F**) loading plot of OPLS-DA(VIP > 2 in red).

The data were imported into SIMCA for data analysis, and the PCA model was exported for unsupervised PCA and HCA analysis. As shown in Figure 3, the model, R^2^X (cum) = 0.678, had two principal components: the first principal component fits the most information and explains 67.8% of the information in the data, and the second principal component explains 7.92% of the information in the data. The first two principal components explain 75.72% of the data fitted by the model. Moreover, all the samples fell within the 95% confidence interval, which indicated that in all the experimental samples, there were no outliers, and none of the 24 samples needed to be excluded from the results. From Figure 3A, it was shown that both of tea stems (CK) and under-expanded experimental samples (180 °C) were in the left zone, while moderately expanded (200 °C, 220 °C, 230 °C) and over-expanded (250 °C) experimental samples were concentrated in the right zone. It suggested that there was a significant difference between the samples, with the less-expanded ones clustered in one zone, and the more-expanded ones clustered in the other zone. It could also be seen that tea stems (CK) and the highest expansion (250 °C) were the furthest from each other, indicating that their differences were the greatest. 

The HCA plot presented that the vertical height in the *Y*-axis direction was proportional to the variability between the samples, and there was a significant distinction between the more puffed samples and the less puffed samples, suggesting that 220 °C was a watershed, and that anything above 220 °C was classified as over-puffed. 

Unsupervised PCA and HCA showed that tea stems (CK) and under-puffed (180 °C) were clustered into one group. Moderately puffed (200 °C, 220 °C, 230 °C) and over-puffed (250 °C) were clustered into one group, and the two could be significantly differentiated from each other. After 200 °C, the compounds in the tea stems were transformed after high-temperature roasting. The next step was to continue to explore the differences between the groups by supervised analysis to look for the differential compounds.

Subsequently, supervised PLS-DA and OPLS-DA analyses were carried out. A permutation test was executed to validate the reliability of the OPLS-DA model. After 500 permutations, it was verified that R^2^ = 0.164 and Q^2^ = −0.382. When R^2^ was less than 0.4 and Q^2^ was less than 0.05, the model was proved to be good with no overfitting. Therefore, the subsequent screening of differential metabolites could be carried out with the OPLS-DA model. The patterns observed in the PLS-DA and OPLS-DA models were in concordance with the PCA results (Figure 3). 

After that, the analysis of labeled compounds was performed, and VIP and loading plot could be used to find labeled compounds in the OPLS-DA model. To enhance the comprehension of metabolites making substantial contributions in the PLS-DA model, variable importance in the project (VIP) values of identified compounds were calculated and checked. Variables with VIP values exceeding 1 were generally deemed to play a crucial role in the PLS-DA discrimination process [27]. In this study, 115 compounds exhibited VIP values surpassing 2. The loading plot model was used to screen out the compounds whose error lines crossed the zero point of the axes. It indicated that certain variables had a greater effect on classification; that the further away from the coordinate axis, the greater the effect on classification; and that the closer variables had a direct positive correlation. That is, one variable increased as the other increased and, conversely, decreased as it decreased. A comprehensive screening and identification process yielded a total of 80 differential metabolites. The metabolites were categorized into the following groups: amino acids (5), flavonoids and flavonoid glycosides (2 + 11), phenolic acids (2), catechins (7), alkaloids (3), organic acids (8), glycosides (9), and so on in Table 3.

**Figure 4:**
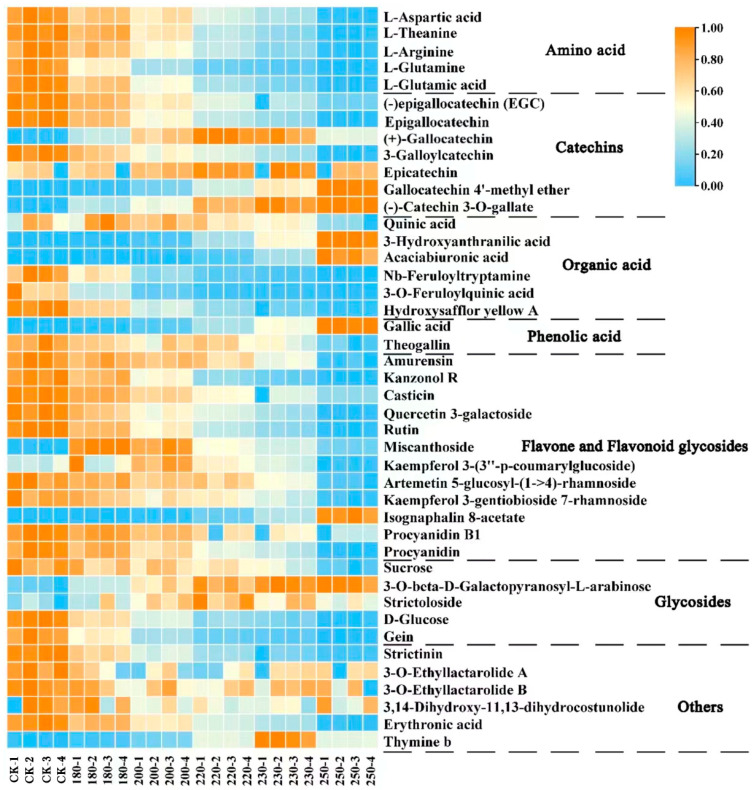
The heatmap of various marker compounds responsible for the classification of different temperature of the tea stems.

In order to show the differential metabolic profiles of tea stems with different degrees of expansion more intuitively, the dynamic changes in metabolites were visualized in this study using heatmaps (Figure 4). The horizontal rows represent the changes in compounds with increasing expansion temperature. The vertical columns represent individual samples. For a sample, blue indicates that the metabolite level was below average and orange indicates that the metabolite level was above average. For a metabolite, blue indicates a negative correlation with expansion temperature, while orange indicates a positive correlation with expansion temperature. The change in labeled compounds with increasing expansion temperature is shown in the heatmap.

There was a great effect on the change in compounds as the expansion temperature increased (Figure 4). Amino acids (*L*-glutamine, *L*-aspartic acid, *L*-glutamic acid, *L*-theanine, and *L*-arginine) gradually decreased with increasing expansion temperature, except for the non-epicatechin catechins (C, GC, GCG, CG). EGC, EC, and EGCG decreased with increasing expansion temperature.

Amino acids constituted the primary source of umami in tea infusion, and the content of amino acids accounted for about 1–4% of the dry weight of tea leaves [28]. *L*-theanine VIP ranked eighth, and its trend was also consistent with the results of the previous report [29,30]. The concentration of some amino acids such as *L*-aspartic acid, *L*-glutamic acid, *L*-glutamine, *L*-theanine, carbohydrates, and epicatechin might be due to the Maillard reaction, oxidative degradation, or isomerization during the roasting process [29,30]. This process leads to the generation of numerous heterocyclic compounds, including pyrazines, pyrroles, and thiazoles [31], which significantly contribute to the sensory qualities of tea stems. For example, *L*-theanine contributes to the formation of roasted and caramelized odors such as pyrazine through a Maillard reaction [24,32]. Also, theanine as the most abundant amino acid in tea had the capability to enhance the umami of tea infusion [4].

The VIP value of EGC was 22.04, which was the highest among all marker compounds and showed a decreasing trend with the increase in expansion temperature. It might be due to the formation of GC by EGC in the high-temperature-induced conditions [31]. The content of quinic acid increased and then decreased during the expansion process. With a VIP value of 14.70, it secured the fourth position, underscoring its significance as a pivotal marker compound. The value of GC increased and then decreased with the increase in temperature. Its VIP value was 13, ranking fifth, which was also the same as the results of Ye et al. [33]. GA gradually increased during the process of expansion temperature. Similar reports had pointed out that gallic acid was most likely to be produced by the degradation of galloylated catechins [34,35], such as EGCG and ECG. Theogallin, rutin, and saccharides (glucose and sucrose) compounds decreased with increasing expansion temperature. Kaneko et al. [36] found that theogallins were compounds that enhance the umami of the tea infusion of green tea. This, to some extent, explained the decrease in the umami of the tea stems with the increase in the expansion temperature. In addition, the results of the study by Xu et al. [37] showed that rutin played a significant contributory role in the astringency of the tea infusion. Sucrose was the predominant form of sugar in tea, accounting for about 0.7% of the dry weight of tea [38]. Its content decreased with increasing expansion temperature, which was in agreement with the results of a previous study. This might be due to the fact that sucrose also participates in the Maillard reaction [28].

Flavonol glycosides could be categorized into three groups according to the glycosidic element: kaempferol glycosides (K-glycosides), myricetin glycosides (M-glycosides), and quercetin glycosides (Q-glycosides) [39]. In this study, most of the flavonoids and flavonol glycosides decreased gradually during the expansion process. The content of flavonol glycosides was changed at different expansion temperatures, which suggested that flavonol glycosides were not stable. This might be due to the fact that flavonol glycosides, as a class of compounds with a low convergence threshold, had their glycosidic bonds broken during heating leading to a change in their content [40,41]. Quercetin 3-galactoside had a VIP value of 4.6, which showed a gradual decreasing tendency with the increase in the expansion temperature. Proanthocyanidins are a class of oligomeric flavonoids consisting of C4 → C6 or C6 → C8 catechins and phenotypic catechins. The proanthocyanidins increased with the increase in expansion temperature in this study. Previous studies have shown that oligomeric proanthocyanidin C1 degraded to form proanthocyanidin B1 at high temperatures and under acidic conditions [31], which explains the result to some extent.

## 4. Conclusions

The quality differences between tea stems under different expansion temperatures were investigated. Meanwhile, the changes in different metabolite levels in tea stems under different expansion gradients were detected. The findings indicate that with the rise in expansion temperature, both the umami and aftertaste of astringency values in tea stems decreased. The content of EGC, EGCG, EC, and tea polyphenols decreased, while the content of amino acids and most of the flavonoids was also decreased. The aftertaste of bitterness of tea stems increased, the content of GA and C increased, and the content of ECG, GC, and GCG showed a tendency to first increase and then decrease. These results showed that EGC was the differential compound with the highest VIP value in different expansion temperature gradients. Quinic acid, GC, and GA were also the key marker difference compounds of tea stems under different expansion temperature conditions. This study not only identified the variation rules and differential compounds of the quality of tea stems at different expansion temperatures, but also provided a corresponding reference for future research on tea stems, which was of great significance for enhancing the utilization value of tea stems.

## Figures and Tables

**Table 1:** Electronic tongue results of tea stems with different expansion temperatures.

Samples	Sourness	Sweet	Umami	Bitterness	Aftertaste-B	Astringency	Aftertaste-A	Richness
Tea stems	−30.90 ± 0.03 ^f^	1.16 ± 0.03 ^b^	18.10 ± 0.10 ^a^	1.54 ± 0.03 ^f^	−0.31 ± 0.01 ^e^	−4.94 ± 0.23 ^a^	2.82 ± 0.27 ^a^	11.89 ± 0.29 ^ab^
180 °C	−30.50 ± 0.06 ^e^	1.05 ± 0.05 ^c^	18.01 ± 0.06 ^a^	1.76 ± 0.02 ^e^	−0.11 ± 0.06 ^d^	−5.06 ± 0.23 ^ab^	2.82 ± 0.31 ^a^	12.28 ± 0.52 ^a^
200 °C	−29.46 ± 0.03 ^d^	1.06 ± 0.07 ^c^	17.40 ± 0.06 ^b^	1.87 ± 0.05 ^d^	0.04 ± 0.04 ^c^	−5.37 ± 0.23 ^bc^	2.57 ± 0.27 ^ab^	12.23 ± 0.58 ^a^
220 °C	−28.51 ± 0.09 ^c^	1.12 ± 0.01 ^bc^	16.85 ± 0.02 ^c^	2.18 ± 0.04 ^c^	0.30 ± 0.05 ^b^	−5.43 ± 0.20 ^bc^	2.53 ± 0.33 ^ab^	12.02 ± 0.36 ^a^
230 °C	−26.56 ± 0.07 ^b^	1.18 ± 0.04 ^ab^	15.57 ± 0.05 ^d^	2.35 ± 0.08 ^b^	0.37 ± 0.02 ^b^	−5.72 ± 0.23 ^cd^	2.26 ± 0.22 ^bc^	11.08 ± 0.54 ^b^
250 °C	−25.70 ± 0.04 ^a^	1.26 ± 0.04 ^a^	14.78 ± 0.02 ^e^	2.86 ± 0.08 ^a^	0.90 ± 0.04 ^a^	−5.85 ± 0.11 ^d^	1.96 ± 0.15 ^c^	9.91 ± 0.48 ^c^

Note: The data are shown as mean ± standard deviation (*n* = 3). Different lowercase letters marked on the same row represent statistically significant differences in the data (*p* < 0.05).

**Table 2:** The routine index measurements results of tea stems with different expansion temperatures.

	Tea Stems	180 °C	200 °C	220 °C	230 °C	250 °C
GA	0.10 ± 0.01 ^f^	0.12 ± 0.00 ^e^	0.21 ± 0.01 ^d^	0.56 ± 0.01 ^c^	0.99 ± 0.01 ^b^	1.87 ± 0.01 ^a^
GC	3.54 ± 0.11 ^d^	4.22 ± 0.12 ^c^	5.00 ± 0.09 ^a^	4.59 ± 0.12 ^b^	4.50 ± 0.08 ^b^	4.46 ± 0.05 ^d^
EGC	31.40 ± 0.68 ^f^	20.75 ± 0.19 ^e^	16.00 ± 0.92 ^d^	11.52 ± 0.31 ^c^	10.04 ± 0.08 ^b^	6.29 ± 0.16 ^a^
C	2.81 ± 0.02 ^d^	3.23 ± 0.04 ^c^	3.60 ± 0.31 ^b^	3.97 ± 0.14 ^a^	3.88 ± 0.04 ^a^	3.96 ± 0.09 ^a^
CAF	16.12 ± 0.43 ^a^	13.21 ± 0.34 ^b^	12.19 ± 0.39 ^d^	12.49 ± 0.29 ^cd^	13.03 ± 0.16 ^bc^	12.41 ± 0.14 ^d^
EGCG	6.19 ± 0.81 ^a^	3.33 ± 0.22 ^b^	2.90 ± 0.32 ^b^	1.53 ± 0.01 ^c^	1.53 ± 0.00 ^c^	1.53 ± 0.00 ^c^
EC	10.27 ± 0.33 ^f^	7.96 ± 0.13 ^e^	6.76 ± 0.30 ^d^	6.01 ± 0.31 ^c^	3.68 ± 0.10 ^b^	2.51 ± 0.04 ^a^
GCG	1.34 ± 0.01 ^b^	1.32 ± 0.02 ^b^	1.41 ± 0.04 ^a^	1.36 ± 0.02 ^b^	1.34 ± 0.00 ^b^	1.13 ± 0.04 ^c^
ECG	1.56 ± 0.12 ^cd^	1.68 ± 0.01 ^ab^	1.78 ± 0.08 ^a^	1.67 ± 0.02 ^bc^	1.66 ± 0.01 ^bc^	1.47 ± 0.01 ^d^
TPs	12.10 ± 0.03 ^a^	11.98 ± 0.44 ^ab^	11.80 ± 0.34 ^ab^	11.68 ± 0.70 ^ab^	11.27 ± 1.02 ^ab^	10.74 ± 0.89 ^b^

Note: The data are shown as mean ± standard deviation (*n* = 3) and compounds (GA, GC, EGC, C, CAF, EGCG, EC, GCG, GLC, NGLC) concentrations are in mg/g. TPs concentrations are in percentage. GA, gallic acid; GC, (−)-gallocatechin; EGC, (−)-epigallocatechin; C, (+)-catechin; CAF, caffeine; EGCG, (−)-epigallocatechin gallate; EC, (−)-epicatechin; GCG, (−)-gallocatechin gallate; TPs, total polyphenols. Different lowercase letters marked on the same row represent statistically significant differences in the data (*p* < 0.05).

**Table 3:** Identification of differential metabolites in tea stems with different expansion temperature.

Var ID	Rt (min)	*m*/*z*	VIP	MS	MS/MS	Identification
8045	6.09	305.067	22.0408	M-H	95.0502, 111.0453, 125.0244, 165.0194	(−)-Epigallocatechin (EGC) ^a^
8044	6.06	305.067	21.6835	M-H	83.0141, 92.5193, 123.009	Epigallocatechin ^a^
6332	20.30	265.148	17.6056	M-H	79.9577, 76.0418	Alkhanol ^b,c^
3534	1.48	191.056	14.6978	M-H	57.346, 85.0297, 111.0454, 146.988, 173.0457	Quinic acid ^a^
8049	5.31	305.067	13.0017	M-H	74.9046, 85.0297, 111.0454	(+)-Gallocatechin ^a^
16562	1.49	533.173	8.9100	M-H	85.0297, 74.9046, 111.0454	Amurensin ^b^
13437	12.47	441.083	8.7218	M-H	57.0346, 109.0296, 165.03	3-Galloylcatechin ^a^
2630	2.21	173.093	8.3926	M-H	58.0298, 74.0249, 112.077, 128.0354, 155.0828	*L*-Theanine ^a^
7295	6.70	289.072	7.7501	M-H	97.0297, 123.0456, 146.9615, 203.0708	Epicatechin ^a^
18446	6.09	611.14	7.6688	M-H	111.0451, 75.8158	Hydroxysafflor yellow A ^b,c^
2629	1.55	173.093	7.5065	M-H	74.0249, 112.077, 128.0357	*L*-Arginine ^a^
2459	4.40	169.014	6.9114	M-H	79.019, 107.0138	Gallic acid ^a^
1476	1.30	145.062	6.2950	M-H	74.0249, 67.0303, 99.0566	*L*-Glutamine ^a^
9618	1.51	341.109	5.7920	M-H	59.0139	Sucrose ^b,c^
10623	2.21	369.176	5.6585	M-H	74.0248, 128.0355, 155.0828	Kanzonol R ^b^
8379	1.29	311.099	5.5857	M-H	59.0139, 97.0296, 113.0246, 161.0455, 177.0414	3-O-beta-d-Galactopyranosyl-l-Arabinose ^bc^
8258	22.20	309.174	5.0432	M-H	74.713, 95.9524	3-O-Ethyllactarolide A ^b^
10734	6.09	373.054	5.0430	M-H	123.0092, 165.0197, 219.0663	Casticin ^b^
10735	6.06	373.054	4.9854	M-H	95.0501, 123.009, 167.0352, 261.0776	Gossypetin 7-methyl ether 8-acetate ^b^
8246	22.16	309.174	4.8889	M-H	95.9524	3-O-Ethyllactarolide B ^b^
14098	1.30	457.168	4.6669	M-H	74.0246, 84.0457, 101.0721, 127.0515	Gein ^b^
14298	12.47	463.065	4.6037	M-H	76.6481, 123.0455, 163.0396, 293.0444	Quercetin 3-galactoside ^b,c^
12073	1.48	405.102	4.5680	M-H	83.0507, 171.03	Strictoloside ^b^
8701	13.08	319.082	4.5427	M-H	81.4057, 109.166, 141.291	Gallocatechin 4′-methyl ether ^b,c^
10513	1.38	367.105	4.1771	M-H	59.014, 71.014, 101.0245, 157.0316	3-O-Feruloylquinic acid ^b^
6333	22.18	265.148	4.0013	M-H	79.9576	Alkhanol ^b,c^
9064	6.09	327.048	3.6648	M-H	77.9093, 124.0165, 161.0248, 218.0578, 242.0273	Aflatoxin G ^b,c^
10736	5.31	373.054	3.6037	M-H	93.0346, 124.0165, 204.0425	Melinervin ^b^
9065	6.06	327.048	3.6010	M-H	69.0347, 125.0246, 161.024, 236.0703	Aflatoxin Q1 ^b,c^
18405	5.44	609.125	3.5650	M-H	109.0299, 165.0194, 283.025	Rutin ^bc^
9681	4.57	343.067	3.5055	M-H	71.0143, 127.0404	Theogallin ^b,c^
1491	1.34	146.046	3.5044	M-H	93.0774, 128.0356	*L*-Glutamic acid ^a^
2330	1.37	165.04	3.4697	M-H	59.0139, 75.0089, 99.0088, 147.0302	1-Methylxanthine ^b,c^
3697	1.35	195.051	3.3607	M-H	72.9932, 129.0196	Gluconic acid ^b,c^
892	1.31	132.03	3.3566	M-H	64.6975, 71.0139	*L*-Aspartic acid ^a^
13438	12.74	441.083	3.3432	M-H	109.0298, 125.0246, 168.0063, 203.0717	(−)-Catechin 3-O-gallate ^a^
8702	13.26	319.082	3.2423	M-H	93.0346, 117.0348	4-Coumaroylshikimic acid ^b,c^
20603	12.95	755.204	3.1390	M-H	211.0401, 239.0346, 284.004	Kaempferol 3-gentiobioside 7-rhamnoside ^b,c^
6342	19.99	265.148	3.0690	M-H	69.1848, 96.9603, 203.445	(6beta,8betaOH)-6,8-Dihydroxy- 7(11)-eremophilen-12,8-olide ^b^
10174	1.29	357.104	3.0537	M-H	61.2803, 101.0248, 125.0245, 161.0458	Uknown
5422	1.56	241.081	2.9387	M-H	74.0249, 82.03, 99.0416, 112.0767, 129.1037	Uknown
9783	1.32	347.075	2.9380	M-H	105.0975, 179.0558	3,5,7,3′,4′,5′-Hexahydroxy- 6,8-Dimethylflavanone ^b^
18890	6.09	633.122	2.8995	M-H	83.014, 125.0246, 161.0245, 259.0592, 305.0666	Strictinin ^b^
18031	6.03	593.13	2.8294	M-H	125.0245	Kaempferol 3-(3″-p-coumarylglucoside) ^b,c^
10091	1.57	355.084	2.8164	M-H	66.7102, 87.0089, 199.0413	Acaciabiuronic acid ^b^
11863	12.54	400.14	2.8072	M-H	109.0296, 203.0722	1,1-dioxo-2-[4-[4-(2-pyrimidinyl)-1-piperazinyl]butyl]-1,2-benzothiazol-3-one ^b^
992	1.52	133.014	2.7314	M-H	59.0138, 92.5198	*L*-Malic acid ^b,c^
2976	1.51	179.056	2.7302	M-H	59.0139, 75.0088, 125.024	D-Glucose ^b,c^
1747	7.16	152.036	2.6819	M-H	68.3053	3-Hydroxyanthranilic acid ^b^
1114	1.42	135.03	2.6153	M-H	59.014, 71.014, 117.0195	Erythronic acid ^b^
10413	1.51	365.109	2.5703	M-H	71.0139, 156.5351	UNPD88418 ^b^
8273	309.21	2.48141	2.6814	17.11	77.1975, 92.5161, 165.1279, 185.1185	Uknown
17389	12.80	568.146	2.4785	M-H	81.0347, 125.0246, 168.1035, 219.0663	(+)-Lactonamycin; Lactonamycin ^b^
19884	1.50	695.225	2.4615	M-H	71.0139, 171.03, 191.0562	Artemetin 5-glucosyl-(1->4)-rhamnoside ^b^
10092	2.20	355.084	2.4584	M-H	87.0089, 152.0205, 196.0102	Isognaphalin 8-acetate ^b^
6349	22.66	265.148	2.4518	M-H	59.9711, 95.9526, 220.812	(6beta,8betaOH)-6,8-Dihydroxy- 7(11)-eremophilen-12,8-olide ^b^
4406	1.35	215.033	2.4457	M-H	84.8719	Methoxsalen ^b^
10624	1.56	369.176	2.4367	M-H	74.0249, 128.0356, 143.514	4-[10-Hydroxygeranyloxy]-5- methylcoumarin acetate ^b^
10406	4.57	365.049	2.4331	M-H	85.0295, 157.051, 169.0143	Garcimangosone D ^b^
8878	1.51	323.029	2.4258	M-H	59.0139, 138.9801	Uridine 5′-monophosphate ^b^
8405	18.66	311.202	2.3780	M-H	127.485, 133.0658	Oseltamivir ^b^
13754	5.40	449.109	2.3611	M-H	95.0504, 181.0349, 223.0616	Miscanthoside ^b^
7693	20.51	297.153	2.3376	M-H	62.6178, 119.0503, 170.0049	Gravelliferone ^b^
1849	2.21	155.083	2.3236	M-H	81.7132, 96.9602, 127.0516	4-Hydroxynonenal ^b^
643	4.41	125.025	2.2770	M-H	108.4477	Thymine ^b,c^
4557	2.19	219.051	2.2461	M-H	78.9593, 96.9604	4-(3-ethylthiophen-2-yl)benzene-1,2-diol ^b^
10029	20.89	353.201	2.2267	M-H	96.9602	Uknown
9066	5.31	327.048	2.2160	M-H	58.959, 83.014, 125.0247, 241.0473	Aflatoxin G bc
17636	6.37	577.136	2.2132	M-H	82.6876, 109.0296, 233.6426	Procyanidin B1 ^b,c^
6231	1.48	262.056	2.1961	M-H	70.03, 85.0297, 111.0452, 129.0196	Ascorbalamic acid ^b,c^
8148	1.35	307.115	2.1456	M-H	78.9591, 100.0404, 109.0409, 128.0356	O-(N-acetyl-alpha-d-galactosaminyl)-l-serine c ^b^
9608	6.07	341.043	2.1417	M-H	55.8406, 125.0247, 239.704	6-(2H-1,3-benzodioxole-5-carbonyloxy)- 3,4,5-Trihydroxyoxane-2-carboxylic acid ^b^
17628	6.33	577.135	2.1223	M-H	109.0294, 151.0401, 245.0819	Procyanidin ^b,c^
10362	6.06	363.025	2.1078	M-H	81.0349, 123.009, 179.0349, 261.0768	Xanthylic acid ^b^
7919	4.19	302.088	2.0658	M-H	87.0091, 128.0354	Gynocardin ^b^
17391	12.30	568.146	2.0616	M-H	91.5343, 169.0143	(+)-Lactonamycin;Lactonamycin ^b^
9401	1.54	335.146	2.0615	M-H	84.0456, 128.0358, 141.1036, 173.0932	Nb-Feruloyltryptamine ^b^
7329	1.62	290.088	2.0389	M-H	84.0456, 101.0245	Sarmentosin epoxide ^b^
4220	1.50	209.067	2.0283	M-H	55.0188, 73.0296, 85.0296, 115.0403	1,3,7-Trimethyluric acid ^b,c^
6214	6.09	261.077	2.0005	M-H	81.0347, 109.0298, 201.056	9,10-dihydroxy-8,8-dimethyl-2H, H,9H,10H-pyrano[2,3-h] chromen-2-one ^b^

^a^ The compounds identified using authentic standards denoted “a”. ^b^ The compounds identified by the analysis software MS-finder (version 3.04) and MS-dial (version 3.82) with mass fragment ions denoted “b”. ^c^ The compounds searched from the public databases such as UNDP, Tea Metabolome databases, and PubChem by MS^2^ spectra denoted “c”.

## Data Availability

Data are contained within the article.
